# Segmentation and classification of colon glands with deep convolutional neural networks and total variation regularization

**DOI:** 10.7717/peerj.3874

**Published:** 2017-10-03

**Authors:** Philipp Kainz, Michael Pfeiffer, Martin Urschler

**Affiliations:** 1 Institute of Biophysics, Center for Physiological Medicine, Medical University of Graz, Graz, Austria; 2 Institute of Neuroinformatics, University of Zurich and ETH Zurich, Zurich, Switzerland; 3 Ludwig Boltzmann Institute for Clinical Forensic Imaging, Graz, Austria; 4 Institute for Computer Graphics and Vision, Graz University of Technology, Graz, Austria; 5 BioTechMed-Graz, Graz, Austria

**Keywords:** Colon glands, Deep learning, Segmentation, Malignancy classification

## Abstract

Segmentation of histopathology sections is a necessary preprocessing step for digital pathology. Due to the large variability of biological tissue, machine learning techniques have shown superior performance over conventional image processing methods. Here we present our deep neural network-based approach for segmentation and classification of glands in tissue of benign and malignant colorectal cancer, which was developed to participate in the *GlaS@MICCAI2015* colon gland segmentation challenge. We use two distinct deep convolutional neural networks (CNN) for pixel-wise classification of Hematoxylin-Eosin stained images. While the first classifier separates glands from background, the second classifier identifies gland-separating structures. In a subsequent step, a figure-ground segmentation based on weighted total variation produces the final segmentation result by regularizing the CNN predictions. We present both quantitative and qualitative segmentation results on the recently released and publicly available Warwick-QU colon adenocarcinoma dataset associated with the *GlaS@MICCAI2015* challenge and compare our approach to the simultaneously developed other approaches that participated in the same challenge. On two test sets, we demonstrate our segmentation performance and show that we achieve a tissue classification accuracy of 98% and 95%, making use of the inherent capability of our system to distinguish between benign and malignant tissue. Our results show that deep learning approaches can yield highly accurate and reproducible results for biomedical image analysis, with the potential to significantly improve the quality and speed of medical diagnoses.

## Introduction

The variability of structures in biological tissue poses a challenge to both manual and automated analysis of histopathology slides (McCann et al., 2015). In recent years automated analysis has become a key requirement for quantitative morphology assessment and cancer grading, since tissue specimens were digitized using whole slide scanners producing gigapixel images. Virtual microscopy already plays an important role in pathology departments, but the problem of intra- and inter-observer variability still remains due to the qualitative inspections of the slides. Although Andrion et al. (1995) showed moderate to good agreement among five expert pathologists, and satisfactory results on their intra-observer reliability, other studies such as Thomas et al. (1983) or more recently Constantini et al. (2003) and Van Putten et al. (2011) found that even experienced pathologists frequently disagree on tissue classification, which may lead to the conclusion that solely using expert scoring as gold standard for histopathological assessment could be insufficient (Aeffner et al., in press). Hence, there is a growing demand for robust computational methods in order to increase reproducibility of diagnoses (Gurcan et al., 2009; Dundar et al., 2011; McCann et al., 2015).

Colorectal cancer is among the leading causes of cancer-related death in developed countries (Torre et al., 2015). Accurate tumor grading is essential for patient survival and can be done most effectively in stained histopathological sections harvested via biopsy or during surgery. Fleming et al. (2012) showed that aggressiveness of colon cancer is reflected by the formation and architecture of glands. In order to allow reliable classification of different tumor types, it is thus highly important to accurately segment glands from other structures in a first step.

**Figure 1:**
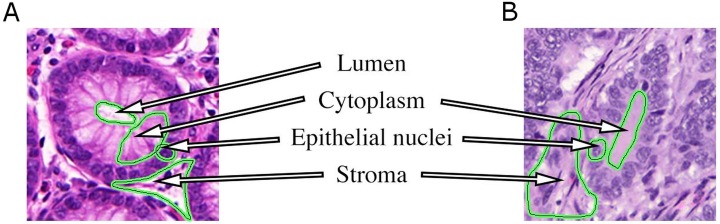
Tissue components in histopathological sections of colon glands, stained with Hematoxylin-Eosin. A benign gland (A) and a malignant gland (B), consisting of the lumen, cytoplasm and epithelial nuclei, which form the gland border. Stroma is not considered part of a gland and contains blood vessels, nervous and connective tissue. In some (especially malignant) cases, the gland lumen may be occluded.

A typical histopathological image of colon glands contains four tissue components: lumen, cytoplasm, epithelial cells, and stroma (connective tissue, blood vessels, nervous tissue, etc.). The epithelial cells form the gland boundary, enclosing cytoplasm and lumen, whereas stroma is not considered part of the gland, see Fig. 1. If we just consider non-cancerous (benign) glands, automated segmentation algorithms must already be able to deal with significant variability in shape, size, location, texture and staining of glands. Moreover, in cancerous cases gland objects can significantly differ from benign glands, and the presence of corrupted areas (artifacts) further exacerbates the segmentation problem. Therefore, machine learning based approaches are predominantly used to learn robust models from labeled examples in order to cope with tissue variability.

### Objectives and organization of this paper

In this work, we present our deep learning-based strategy for the segmentation of glands and classification of benign and malignant tissue, which was developed to participate in the *GlaS@MICCAI2015* colon gland segmentation challenge. The contributions of our work are twofold: driven by the lack of huge numbers of input training images, we firstly present a deep learning scheme to generate classifier predictions that distinguishes gland and background pixels for malignant and benign tissues. This is accompanied in our design by a dedicated gland-separating refinement classifier that is able to separate touching objects, which pose a challenge for later figure-ground segmentation. Secondly, we use these classification results as the input for a simple, yet effective, globally optimal figure-ground segmentation approach based on a convex geodesic active contour formulation that regularizes the classifier predictions according to a minimal contour-length principle by involving total variation (TV) regularization. Moreover, our approach is inherently able to distinguish benign and malignant tissue due to a convenient formulation of the semantic pixel-labeling problem. Both steps are described in ‘Methods’, while subsequent sections show and discuss the results of applying our approach to the recently released *Warwick-QU* dataset (Sirinukunwattana, Snead & Rajpoot, 2015) containing Hematoxylin-Eosin (H&E) stained sections of colorectal cancer, which is the target of the *GlaS@MICCAI2015* challenge (Sirinukunwattana et al., 2017).

### Related work

Previous approaches towards gland segmentation and tissue grading in histopathology sections can roughly be categorized into three classes: (i) Low-level methods based on features computed from texture and color intensities, (ii) high-level methods using graphical models, and (iii) hybrid methods that take into account multiple levels of information present in an image. The majority of previous works regard colon and prostate tissue and share the idea of first identifying significant visual cues, e.g., gland lumen, which are used as seed regions for subsequent processing steps. Furthermore, the level of prior knowledge included in the approaches also varies significantly. Related methods operate on grey level images, or take advantage of color information represented in the RGB or CIEL*a*b color space.

Wu et al. (2005) addressed the segmentation problem of intestinal glands by working with conventional image processing methods such as thresholding, morphological operations, and seeded region growing (SRG), using a significant level of prior knowledge on the structure of typical glands. In Farjam et al. (2007) a first feature space from grey value images was constructed, and a second one from variance-filtered images using textural features. Tissue components were identified by employing *k*-means clustering on the first feature space to separate stroma/lumina (as one class) from the rest of the image, and on the second feature space to separate nuclei from the rest of the image. Finally, clustering results were combined by excluding nuclei from stroma and lumina to obtain the glandular regions. Subsequently, glands were classified into benign and malignant using a linear classifier. A different approach was pursued by Naik et al. (2008), where they trained a Bayesian classifier to generate pixel-wise probability maps for lumen, cytoplasm and nuclei. Prior knowledge of the gland size and structure was estimated from the training set and used to remove “false” lumen candidates. A level-set approach segments glands based on the probabilities for gland border nuclei. A high-throughput system for detecting prostate cancer was built by Monaco et al. (2010). They first identified gland centers as maxima in a Gaussian scale space, and then employed probabilistic pairwise Markov models and SRG to delineate the gland borders. Candidate glands were then classified and malignant glands were consolidated into cancerous regions. The work of Peng et al. (2011) relied on *k*-means clustering, morphological operations, and SRG to segment the glands. Linear discriminant analysis (LDA) was used to distinguish between benign and malignant glands. In a more recent paper (Fu et al., 2014), a gland detection and segmentation approach named “GlandVision” was proposed. A random field model was employed to locate candidate gland boundaries in a polar coordinate-transformed image. Candidate gland boundaries were verified by a support vector regressor. A requirement of this approach is that gland borders need to be fully intact, which limits their applicability on images in the *Warwick-QU* dataset. However, they showed good performance on H&E and Hematoxylin-Diaminobenzidine (H-DAB) stained tissue.

The common ideas of graphical high-level models for colon gland segmentation and classification are the representation of relations among tissue components as graphs, and modeling the regular structure of a gland (see also Fig. 1). Rather than working at the pixel level directly, tissue components are first identified by clustering the intensity space using *k*-means, and then locally approximated by circular primitives. Centroids of the primitives represent nodes in an undirected object-graph, where the nodes are labeled by *k*-means as gland or non-gland, depending on their local spatial relationship. Nodes were subsequently used as seed points for applying SRG on the graph and producing a segmentation (Gunduz-Demir et al., 2010; Tosun & Gunduz-Demir, 2011), followed by learning decision trees to eliminate false glands (Gunduz-Demir et al., 2010). A set of structural features could then be extracted to diagnose and grade colorectal cancer (Altunbay et al., 2009). Sirinukunwattana, Snead & Rajpoot (2015) recently proposed a random polygon model for the segmentation of colon adenocarcinoma. A gland is modeled as a polygon with a random number of vertices that are approximately located on gland border nuclei. First, gland lumen are identified by classifying superpixel features using a random decision forest, followed by identifying border nuclei and constructing the polygons from a set of seed areas. A postprocessing step ensures elimination of weak hypotheses and smooth gland boundaries.

A method for grading prostate cancer that explored the efficacy of textural and morphological features in addition to tissue architecture was presented by Doyle et al. (2007). The identified texture features are the most important features to contribute to reliable tissue grading. Nguyen, Sarkar & Jain (2012) integrated low-level and contextual features in a prostate gland segmentation algorithm. It was based on the association of endothelial nuclei to lumen, and as first step performs *k*-means clustering in the RGB color space to label four tissue components in the image. A connected component analysis further revealed lumen and nuclei blobs, and a convex hull enclosing the border nuclei is considered the segmentation result. Furthermore, classification into benign and malignant glands as well as artifacts was performed, using structural context information (Nguyen, Sarkar & Jain, 2012). Rashid et al. (2013) extended this concept by using LDA as pixel-classifier on local image patches in CIEL*a*b color space to predict the four tissue components. Nuclei object candidates were generated by the watershed algorithm. Non-nuclei objects were rejected using a support vector machine, before the final segmentation was created according to the method of Nguyen, Sarkar & Jain (2012). Classification into malignant and benign was performed by empirically estimating proper thresholds on two novel features.

Recently, deep learning methods, especially convolutional neural networks (CNNs) (LeCun, Kavukcuoglu & Farabet, 2010), have received substantial attention in the medical imaging domain. They have found applications in biomedical image analysis for tasks such as semantic segmentation (Pang et al., 2010; Long, Shelhamer & Darrell, 2015; Ronneberger, Fischer & Brox, 2015), mitosis detection and classification (Cireşan et al., 2013; Malon & Cosatto, 2013), and blood cell counting (Habibzadeh, Krzyżak & Fevens, 2013). To the best of our knowledge, deep learning methods have not been proposed for gland segmentation and classification before the *GlaS@MICCAI2015* challenge. The most successful methods within the challenge were mostly based on deep learning, and an overview of the competing methods can be found in Sirinukunwattana et al. (2017).

## Methods

We present a segmentation method for H&E stained histopathological sections that proceeds in three steps: (1) The raw RGB images are preprocessed to extract a normalized representation of the tissue structure; (2) Two pixel-wise classifiers are trained that distinguish glands from background (*Object-Net*), and identify gland-separating structures (*Separator-Net*) in the image; (3) The outputs of the classifiers are combined and a figure-ground segmentation based on weighted total variation (wTV) is used to produce the segmentation result. Due to restrictions in training dataset size, we decided to separate this task into several steps, namely a classifier, a separator and a regularizer that smoothes the final segmentation result. In the following sections, the three steps of our method are explained in more detail. We further show that the proposed approach is able to classify images of benign and malignant tissue as a side product without any additional computational cost.

**Figure 2:**
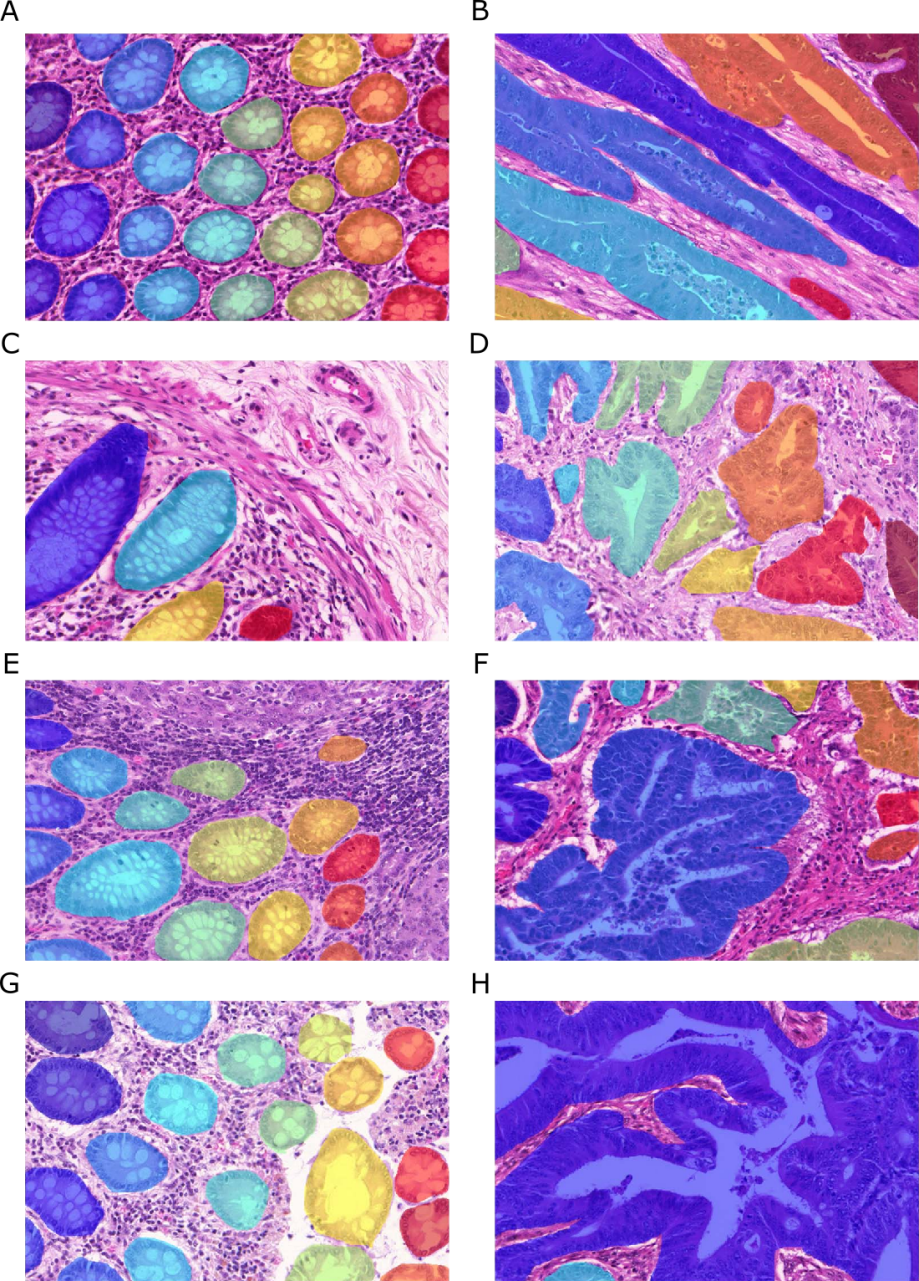
Samples of benign (A, C, E, G) and malignant (B, D, F, H) colorectal cancer sections in the *Warwick-QU* dataset. Ground truth labels in each image are available for each pixel and overlaid in different colors for individual objects, while background is transparent. Benign and malignant glands vary greatly in size, shape, and texture making this dataset challenging.

### Dataset

Our method is evaluated on the recently released *Warwick-QU* dataset (Sirinukunwattana, Snead & Rajpoot, 2015), which is the target of the *GlaS@MICCAI2015* challenge and publicly available from the contest website (http://www.warwick.ac.uk/bialab/GlasContest). The dataset contains 165 annotated images of benign and malignant colorectal adenocarcinoma, stained with H&E and scanned at 20 × magnification using a Zeiss MIRAX MIDI Scanner. Pixel resolution of the images was isotropic at 0.62 µm.

Ground truth annotations were provided as images, where the background pixels are labeled zero, and pixels belonging to individual gland objects were labeled with non-zero integer values. Figure 2 shows example images and their ground truth annotations. In each image, all pixels of individual glands are annotated with the same label, illustrated by the different colors, while background is transparent. To the challenge participants, information on whether an image shows benign or malignant tissue is only available in the training dataset. Three datasets were released during the contest and the total number of non-overlapping images (benign/malignant) in the training set, test set A and test set B is 85 (37/48), 60 (33/27), and 20 (16/4), respectively. All three datasets come from the *Warwick-QU* collection, thus they were stained in the same center and digitized using the same scanner. The datasets contained 795, 666, and 95 individual glands.

### Preprocessing H&E slides

**Figure 3:**
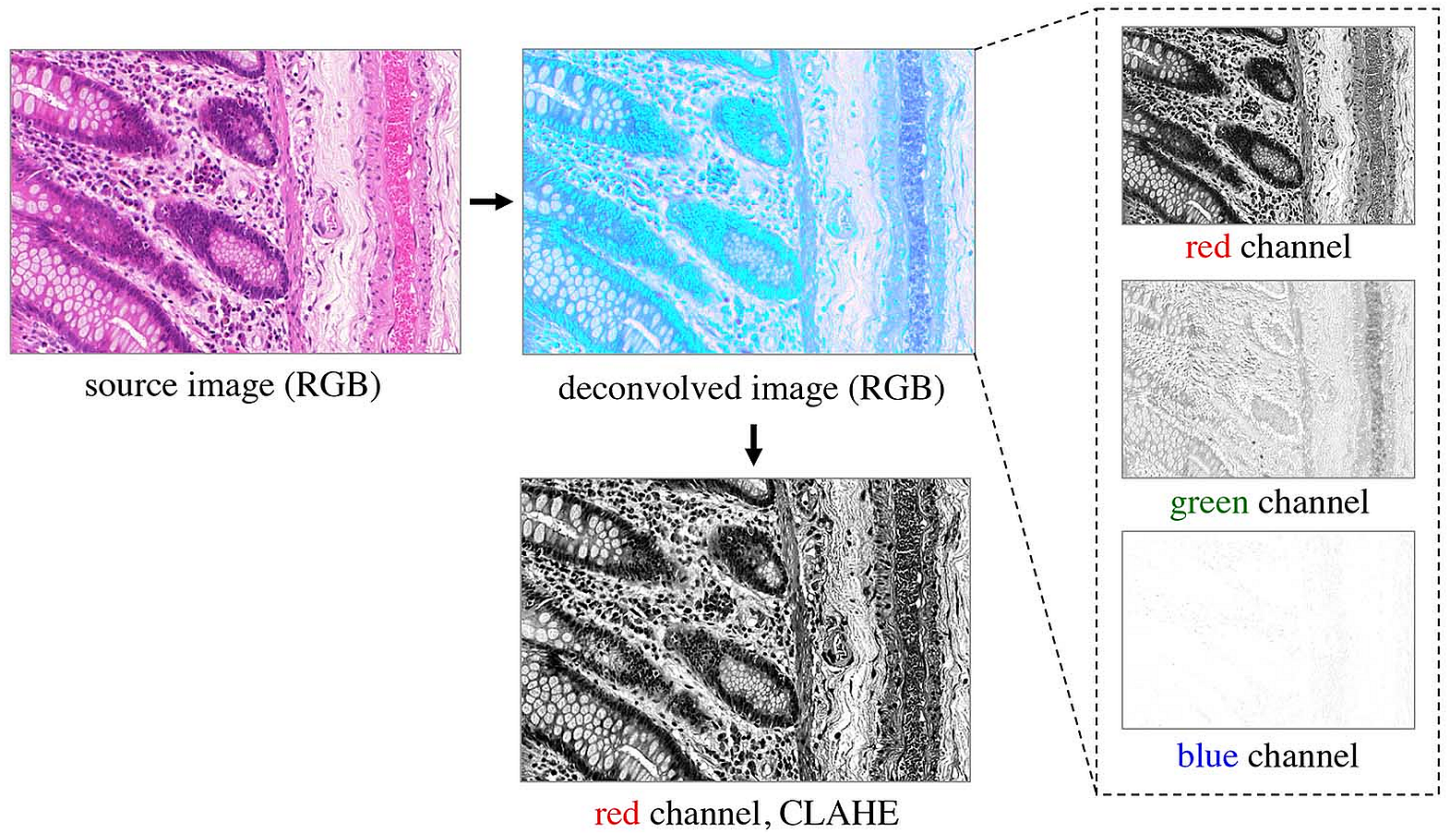
Preprocessing of the RGB images. Color deconvolution (Ruifrok & Johnston, 2001) separates the H&E stained tissue components. Considering the deconvolved image, the green channel expresses very low contrast, and the blue channel does not contain any relevant information on the tissue structure; both are therefore omitted from further processing. The red channel represents most of the tissue structure. It is processed by CLAHE (Zuiderveld, 1994) and taken as input for the pixel classifiers.

Prior to classification, the RGB images are preprocessed as shown in Fig. 3. A color deconvolution (Ruifrok & Johnston, 2001) is performed for the H&E staining used in the provided dataset. It separates tissue components according to their staining, emphasizes the tissue structure and inherently performs data whitening. We used the “H&E 2” setting in the implementation available in Fiji (Schindelin et al., 2012). The first (red) channel of the deconvolved RGB image contains most of the relevant tissue structure information, so the other channels are omitted. In order to account for different staining contrasts and illumination conditions during image acquisition, contrast limited adaptive histogram equalization (CLAHE, Zuiderveld (1994)) is finally applied to the kept red channel.

### Learning pixel classifiers

Given the large variability of both benign and malignant tissue in the *Warwick-QU* dataset (see Fig. 2), we chose to apply deep convolutional neural network classifiers due to their recently shown convincing performance in complex visual classification problems in general (Krizhevsky, Sutskever & Hinton, 2012; Simonyan & Zisserman, 2014; Szegedy et al., 2015), and pixel-wise classification of histopathology images (Cireşan et al., 2013) in particular. Their advantage lies in their ability to extract rich hierarchies of meaningful features from labeled image datasets.

#### CNN architecture

**Figure 4:**
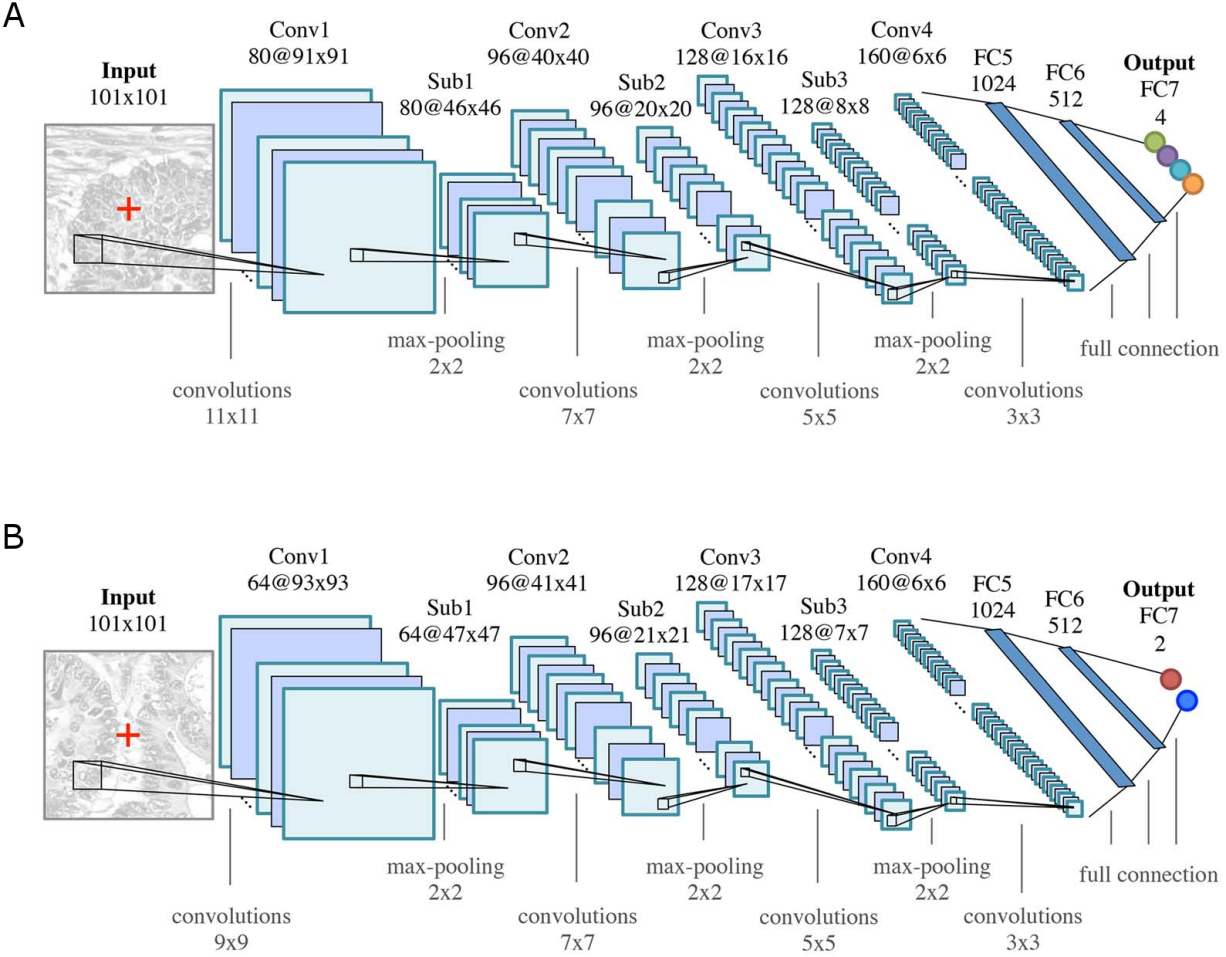
CNN classifier architectures of (A) the *Object-Net* for predicting one of the four segmentation labels and (B) the *Separator-Net* for separating glands. Both architectures have *K* = 7 (*k* = 1, …, *K*) layers. They are identical in the number of convolutional (Conv*k*), max-pooling (Sub*k*), and fully connected (FC*k*) layers, but differ in convolution kernel size, size and number of the feature maps, as well as the number of output units. The CNN predicts the probability distribution over *L* labels of the center pixel **x** = (*u*, *v*)^⊤^ (marked as red cross in the input patch).

The general architecture of both CNNs is motivated by the LeNet-5 architecture (LeCun et al., 1998), and consists of *K* = 7 (*k* = 1, …, *K*) layers: four convolutional layers (Conv *k*) for feature learning and three fully connected (FC*k*) layers as feature classifiers, see Fig. 4. Rectified linear unit (ReLU) nonlinearities (*f*(*x*) = max(0, *x*)) are used as activation functions throughout all layers in the networks. All convolutional layers consist of a set of learnable square 2D filters, followed by ReLU activation. Subsampling (max-pooling) layers (Sub*k*, 2 ×2 ) are used after the first three convolutional layers. The final pixel-wise classification of an input image is obtained by sliding a window of size 101 ×101 pixels over the image, and classifying the center pixel of that window. Differences between the two CNN architectures are due to the smaller field of view that is required for modeling the boundaries between glands, as opposed to the classification into benign/malignant glands or background.

For training, minibatch stochastic gradient descent with momentum, weight decay, and dropout regularization is used to minimize a negative log-likelihood loss function. Training the networks for classification is thus equivalent to minimizing a cross-entropy loss. For a more detailed explanation of this widely used setup and the involved parameters, we refer the reader to (LeCun et al., 1998) and (Goodfellow, Bengio & Courville, 2016).

#### *Object-Net*: classifying gland objects

**Figure 5:**
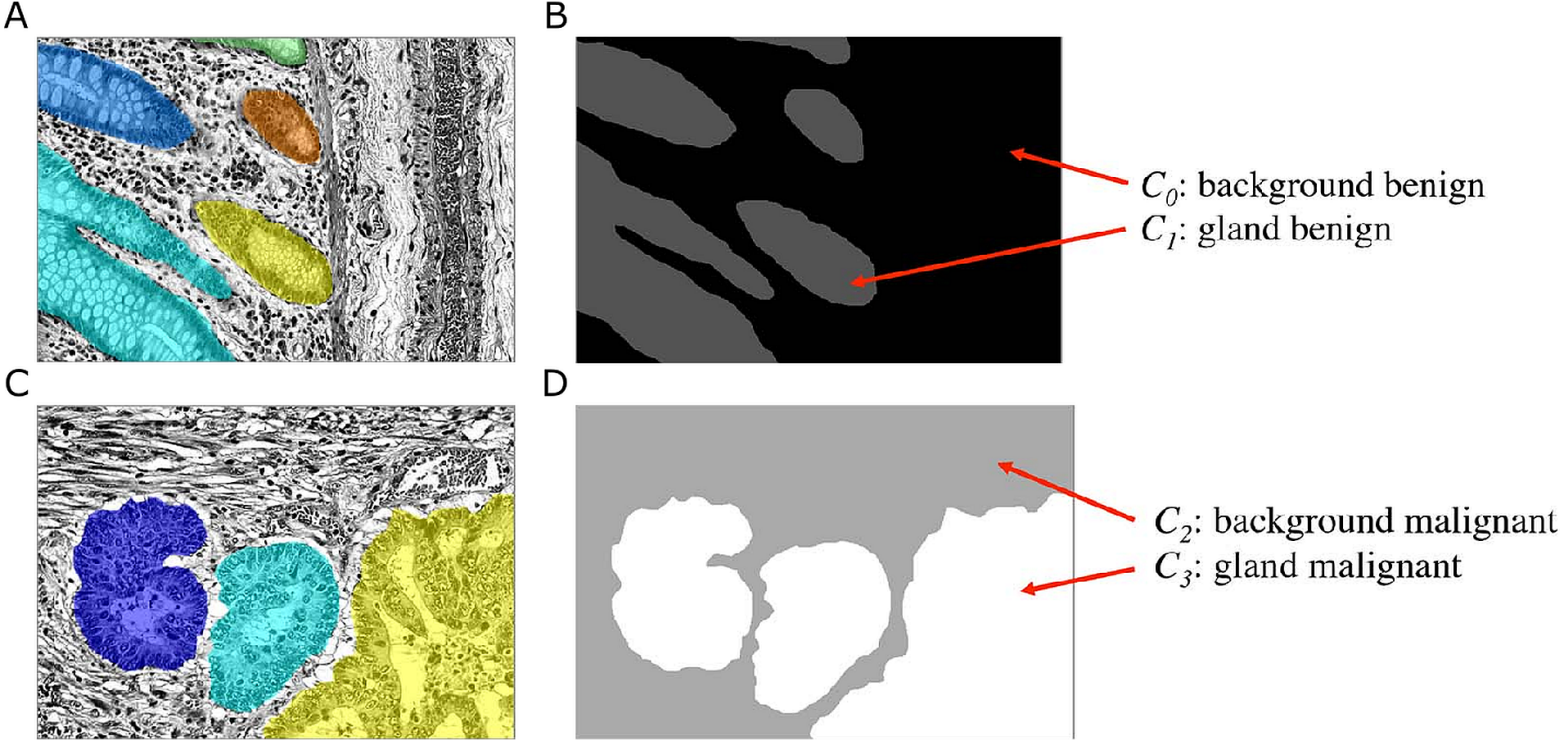
Ground truth label transformation for learning the four-class classification problem on the preprocessed images with the *Object-Net*. (A, B) show a benign case, (C, D) a malignant case. (A, C) Preprocessed images with overlaid individual ground truth object annotations. (B, D) Provided annotations were transformed into four labels for benign background (*C*_0_), benign gland (*C*_1_), malignant background (*C*_2_) and malignant gland (*C*_3_).

The goal of the *Object-Net* is to predict the probability of a pixel belonging either to a gland or to any other tissue structure, i.e., background. Although this could be formulated as a binary classification task, the unique features of benign and malignant tissues, which are not found in the other tissue type, allow a more specific exploitation of these features. We therefore formulate a classification problem, in which we distinguish four classes *C*_*l*_, *l* = {0, …, 3}: benign background (*C*_0_), benign gland (*C*_1_), malignant background (*C*_2_), and malignant gland (*C*_3_). This requires a transformation of the ground truth labels available in the *Warwick-QU* dataset, which now also indicate whether the gland is of benign or malignant type. Hence, a new label is assigned to pixels belonging to each class *C*_*l*_, see Fig. 5.

The input to the CNN is an image patch *I*(**x**) of size 101 × 101 pixels, centered at an image location **x** = (*u*, *v*)^⊤^, where **x** ∈ Ω and Ω denotes the image domain. A given patch *I*(**x**) is convolved with 80 filters (11 × 11) in the first convolutional layer, in the second layer with 96 filters (7 × 7), in the third layer with 128 filters (5 × 5), and in the last layer with 160 filters (3 × 3), see Fig. 4A. The three subsequent fully connected layers FC5-FC7 of the classifier contain 1,024, 512, and four output units, respectively. The output of FC7 is fed into a softmax function, producing the center pixel’s probability distribution over the labels. The probability for each class *l* is stored in a corresponding map *I*_*C*_*l*__(**x**).

#### *Separator-Net*: classifying gland-separating structures

Initial experiments have shown that taking pixel-wise predictions only from the *Object-Net* were insufficient in order to separate very close gland objects. Hence, a second CNN, the *Separator-Net*, is trained to predict structures in the image that separate such objects. This learning problem is formulated as a separate binary classification task using the manually created label annotations defined in ‘Training dataset sampling’.

As depicted in Fig. 4B, the CNN structure is similar to the *Object-Net*: a given input image patch *I*(**x**) of size 101 × 101 pixels is convolved with 64 filters (9 × 9) in the first convolutional layer, in the second layer with 96 filters (7 × 7), in the third layer with 128 filters (5 × 5), and in the last layer with 160 filters (3 × 3). The three subsequent fully connected layers FC5-FC7 of the classifier contain 1,024, 512, and two output units, respectively. The output of the last layer (FC7) is fed into a softmax function to produce the probability distribution over the labels for the center pixel. The probability for a pixel **x** belonging to a gland-separating structure is stored in the corresponding probability map *S*(**x**).

#### Refining *Object-Net* outputs

Once all probability maps have been obtained, the *Object-Net* predictions *I*_*C*_*l*__(**x**) are refined with the *Separator-Net* predictions *S*(**x**) in order to emphasize the gland borders and to prevent merging of close objects. The subsequent figure-ground segmentation algorithm requires a single foreground and background map to produce the final segmentation result, so outputs are combined as follows.

The foreground probability map *p*_*fg*_ is constructed by

(1) \begin{eqnarray*}{p}_{fg}(\mathbf{x})=\max \left\{ \left( \sum _{l\in \{1,3\}}{I}_{{C}_{l}}(\mathbf{x}) \right) -S(\mathbf{x}),0 \right\} ,\end{eqnarray*}

and the background probability map *p*_*bg*_ accordingly:

(2) \begin{eqnarray*}{p}_{bg}(\mathbf{x})=\min \left\{ \left( \sum _{l\in \{0,2\}}{I}_{{C}_{l}}(\mathbf{x}) \right) +S(\mathbf{x}),1 \right\} .\end{eqnarray*}

### Regularization by total variation segmentation

To generate a final segmentation, following continuous non-smooth energy functional *E*_seg_(*u*) (Hammernik et al., 2015) is minimized:

(3) \begin{eqnarray*}\begin{array}{@{}l@{}} \displaystyle \min _{u}{E}_{\mathrm{seg}}(u)=\min _{u}\int \nolimits _{\Omega }g(\mathbf{x}){|}\nabla u(\mathbf{x}){|}\,\mathrm{d}\mathbf{x}+\lambda \int \nolimits _{\Omega }u(\mathbf{x})\cdot w(\mathbf{x})\,\mathrm{d}\mathbf{x}\\ \displaystyle \text{s.t.} u\in {C}_{\mathrm{box}}=\{u:u(\mathbf{x})\in [0,1],\,\forall \,\mathbf{x}\in \Omega \} \end{array}\end{eqnarray*}

where Ω denotes the image domain and *u* ∈ *C*^1^:Ω↦ℝ is smooth. The first term denotes the *g*-weighted total variation (TV) semi-norm which is a relaxation based reformulation of the geodesic active contour energy (Bresson et al., 2007). The edge function *g*(**x**) is defined as

(4) \begin{eqnarray*}g(\mathbf{x})={e}^{-\alpha \parallel \nabla I(\mathbf{x}){\parallel }^{\beta }},\,\alpha ,\beta \gt 0,\end{eqnarray*}

where ∇*I*(**x**) is the gradient of the input image, thus attracting the segmentation towards large gradients. The second term in Eq. (3) is the data term with *w* describing a weighting map. The values in *w* have to be chosen negative if *u* should be foreground and positive if *u* should be background. If values in *w* are set to zero, the pure weighted TV energy is minimized seeking for a minimal contour length segmentation. We use the refined outputs from the previous classification step (Eqs. (1) and (2)) and introduce a threshold *τ* to ensure a minimum class confidence in a map *p*:

(5) \begin{eqnarray*}p(\mathbf{x})= \left\{ \begin{array}{@{}ll@{}} \displaystyle 0 &\displaystyle \text{if} p(\mathbf{x})\lt \tau \\ \displaystyle w(\mathbf{x}) &\displaystyle \mathrm{otherwise} \end{array} \right. .\end{eqnarray*}

The weighting map *w* is derived by applying the logit transformation:

(6) \begin{eqnarray*}w(\mathbf{x})= \left\{ \begin{array}{@{}ll@{}} \displaystyle -(\log \nolimits ({p}_{fg}(\mathbf{x}))-\log \nolimits (1-{p}_{fg}(\mathbf{x}))) &\displaystyle \text{if} {p}_{fg}(\mathbf{x})\gt {p}_{bg}(\mathbf{x})\\ \displaystyle \log \nolimits ({p}_{bg}(\mathbf{x}))-\log \nolimits (1-{p}_{bg}(\mathbf{x})) &\displaystyle \text{if} {p}_{fg}(\mathbf{x})\leq {p}_{bg}(\mathbf{x}) \end{array} \right. .\end{eqnarray*}

The regularization parameter *λ* defines the trade-off between data term and weighted TV semi-norm. The stated convex problem in Eq. (3) can be solved for its global optimum using the primal–dual algorithm (Chambolle & Pock, 2011), which can be implemented very efficiently by benefiting from the parallel computing power of modern GPUs. As the segmentation *u* is continuous, the final segmentation is achieved by thresholding *u* with a value of 0.5. We optimize the free parameters *α*, *β* and *λ* by performing a grid search in a suitable range of these values (*α* ∈ [0.5, 15], *β* ∈ [0.35, 0.95] and *λ* ∈ [0.01, 10]), where all 85 annotated training images are used to tune these parameters based on the pixel-level Dice coefficient (Dice, 1945).

### Tissue classification

In the proposed approach, the *Object-Net* implicitly learns a discrimination of benign (*c* = 0) and malignant (*c* = 1) tissue, since the labels for benign and malignant are available in the training dataset, for which a four-class classification problem was proposed. By combining the maps for benignity and malignancy, the average conditional probabilities for a benign case given the corresponding probability maps can be computed as

(7) \begin{eqnarray*}\overline{\mathrm{P}}(c=0{|}{I}_{{C}_{0}},{I}_{{C}_{1}})= \frac{1}{{|}\Omega {|}} \sum _{\mathbf{x}\in \Omega }{I}_{{C}_{0}}(\mathbf{x})+{I}_{{C}_{1}}(\mathbf{x}),\end{eqnarray*}

and similarly for a malignant case as

(8) \begin{eqnarray*}\overline{\mathrm{P}}(c=1{|}{I}_{{C}_{2}},{I}_{{C}_{3}})= \frac{1}{{|}\Omega {|}} \sum _{\mathbf{x}\in \Omega }{I}_{{C}_{2}}(\mathbf{x})+{I}_{{C}_{3}}(\mathbf{x}),\end{eqnarray*}

where |Ω| is the number of pixels in the image domain Ω. The maximum of both values finally indicates the prediction:

(9) \begin{eqnarray*}{c}^{\ast }={\mathrm{arg} \mathrm{max}}_{c} \left\{ \overline{\mathrm{P}}(c{|}\cdot ,\cdot ) \right\} .\end{eqnarray*}

### Performance evaluation metrics

#### Gland segmentation

Quantitative evaluation metrics were computed for gland detection (F1-score), segmentation overlap (Dice index) and shape similarity (Hausdorff distance) at the object-level. The manually annotated object having the maximum overlap with a segmentation hypothesis is considered the associated ground truth for that segmentation. A minimum area overlap of 50% between them is required to consider a detection as true positive (*TP*), otherwise it is considered a false positive (*FP*). Remaining ground truth objects having less than 50% overlap with a segmentation are counted as false negative (*FN*). The F1-score measures the gland detection performance and is defined by

(10) \begin{eqnarray*}\mathrm{F1}= \frac{2\cdot \mathrm{PRC}\cdot \mathrm{REC}}{\mathrm{PRC}+\mathrm{REC}} ,\end{eqnarray*}

where precision is defined by

(11) \begin{eqnarray*}\mathrm{PRC}= \frac{TP}{TP+FP} \end{eqnarray*}

and recall by

(12) \begin{eqnarray*}\mathrm{REC}= \frac{TP}{TP+FN} .\end{eqnarray*}

The pixel-level segmentation performance is evaluated using the Dice coefficient (Dice, 1945), which measures the overlap between two sets of pixels:

(13) \begin{eqnarray*}\mathrm{Dice}(G,S)= \frac{2\cdot {|}G\cap S{|}}{{|}G{|}+{|}S{|}} ,\end{eqnarray*}

where *G* and *S* denote the set of ground truth and segmented pixels, respectively, and |⋅| denotes the cardinality of a set. The object-level Dice coefficient represents an integrated value for how well ground truth and segmentation, and segmentation and ground truth overlap, respectively. It is defined by

(14) \begin{eqnarray*}{\mathrm{Dice}}_{\mathrm{obj}}(G,S)= \frac{1}{2} \left[ \sum _{i=1}^{{n}_{G}}{\tilde {\omega }}_{i}\mathrm{Dice}({\tilde {G}}_{i},{\tilde {S}}_{i})+\sum _{i=1}^{{n}_{S}}{\omega }_{i}\mathrm{Dice}({G}_{i},{S}_{i}) \right] ,\end{eqnarray*}

where ${\tilde {G}}_{i}$ denotes the set of pixels belonging to the *i*th ground truth object, and ${\tilde {S}}_{i}$ the set of pixels in a segmented object that maximally overlaps with ${\tilde {G}}_{i}$. Conversely, *S*_*i*_ denotes the set of pixels belonging to the *i*th segmented object, and *G*_*i*_ the set of pixels in a ground truth object that maximally overlaps with *S*_*i*_. *n*_*G*_ and *n*_*S*_ are the total numbers of individual ground truth objects and segmented objects, respectively. The weighting terms ${\tilde {\omega }}_{i}={|}{\tilde {G}}_{i}{|}/{\mathop{\sum }\nolimits }_{j=1}^{{n}_{G}}{|}{\tilde {G}}_{j}{|}$ and ${\omega }_{i}={|}{S}_{i}{|}/{\mathop{\sum }\nolimits }_{j=1}^{{n}_{S}}{|}{S}_{j}{|}$ capture the relative area of an object *i* in the respective sets.

Shape similarity is assessed using the Hausdorff distance, which is defined by

(15) \begin{eqnarray*}\mathrm{HD}(G,S)=\max \{{\mathrm{sup}}_{x\in G}\inf _{y\in S}\parallel x-y\parallel ,{\mathrm{sup}}_{y\in S}\inf _{x\in G}\parallel x-y\parallel \}.\end{eqnarray*}

At the object-level, the shape similarity is measured between all segmented objects and all ground truth objects using the object-level Hausdorff distance:

(16) \begin{eqnarray*}{\mathrm{HD}}_{\mathrm{obj}}(G,S)= \frac{1}{2} \left[ \sum _{i=1}^{{n}_{G}}{\tilde {\omega }}_{i}{\mathrm{HD}}_{\mathrm{obj}}({\tilde {G}}_{i},{\tilde {S}}_{i})+\sum _{i=1}^{{n}_{S}}{\omega }_{i}\mathrm{HD}({G}_{i},{S}_{i}) \right] .\end{eqnarray*}

#### Tissue classification

The classification performance for benign and malignant tissue is computed from a 2 × 2 confusion matrix **M** in terms of overall accuracy

(17) \begin{eqnarray*}\mathrm{ACC}= \frac{\mathrm{tr}(\mathbf{M})}{\sum _{i}{\mathbf{M}}_{i}} ,\end{eqnarray*}

where tr(**M**) denotes the trace of the confusion matrix, and *i* the *i*th element of the matrix. Similarly, tissue classification performance is reported class-wise as F1-score, precision, and recall using  Eqs. (10)–(12). Please note that different to the definition given for segmentation above, here we refer to *TP* as true positive, *FP* as false positive, and *FN* as false negative classified *cases*, i.e., entire images.

### Implementation details

#### Training dataset sampling

For the sake of execution speed when using a sliding window approach for pixel-wise classification, the images are rescaled to half resolution prior to classification with the CNNs, and upsampled with bilinear interpolation to their original size before applying the TV segmentation. The size of the input patch *I*(**x**) is chosen to be 101 × 101 pixels, such that sufficient contextual information is available to classify the center pixel.

**Figure 6:**
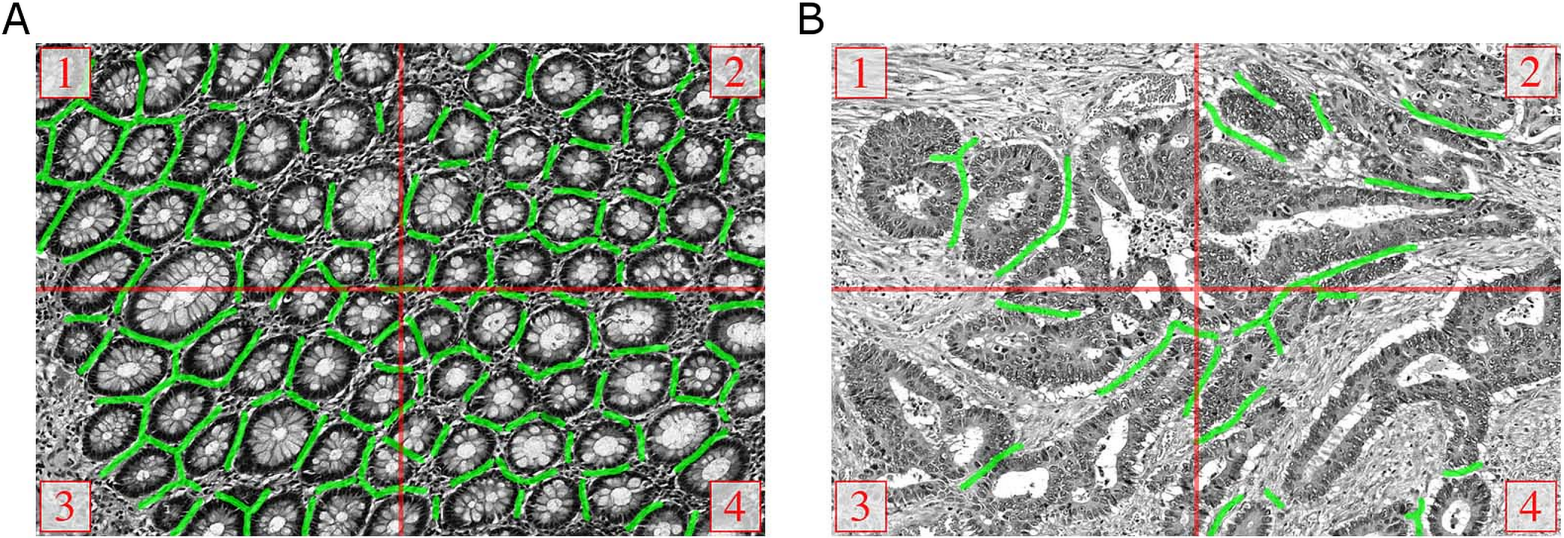
Manual ground truth annotations for gland-separating structures. (A, B) Stitched images from four tiles of two examples from the *GlaS@MICCAI2015* dataset, red lines denote the tile borders. Manual annotations of pixels belonging to gland-separating structures are shown as green lines, the thickness of lines is increased for better illustration.

The majority of training images (79) have a size of 775 × 522 pixels, and rescaling reduces them to 387 ×261 pixels. If only the valid part without border extension would be considered for sampling the patches for the training dataset, approximately 46% of the labeled pixels would be lost when using a patch size of 101 ×101 pixels. On the other hand, a significant number of boundary artifacts would be introduced by artificially extending the border. Fortunately, most images in the training set are tiles of a bigger image and can thus be stitched seamlessly to obtain a total of 19 images (Fig. 6). From these images, enough patches can be sampled without heavily relying on artificial border extension. In one case full stitching was not possible, since only three tiles were available. These three tiles, and the remaining six images that were not part of a bigger image, were treated as individual images.

In principle, the same sampling strategy was pursued for the *Separator-Net*, but it was necessary to create the ground truth labels manually. We annotated all pixels that belong to a structure very close to two or more gland borders as separating structures. The green lines in Fig. 6 illustrate the additional manual annotation of the separating structures (note that the green lines were increased in thickness for the figure to improve visualization). Due to the low number of foreground samples when compared to the *Object-Net*, the number of foreground samples for the *Separator-Net* was artificially increased by exploiting rotation-invariance, and adding nine additional rotated versions of the patch, i.e., every 36°.

#### CNN training

Both CNNs were trained on a balanced training set of 125, 000 image patches per class. Patches in the training sets were sampled at random from the available pool of training images. Training and test sets reflect approximately the same distribution of samples over images. The size of the minibatches in the stochastic gradient descent optimization scheme was set to 200 samples and the networks were trained until the stopping criterion was met: no further improvement of the error rate on a held-out validation set over 20 epochs. We set the initial learning rate to *η*_0_ = 0.0025, with a linear decay saturating at 0.2*η*_0_ after 100 epochs. For all layers, a weight decay was chosen to be 0.005 and the dropout rate was set to 0.5. An adaptive momentum term was used, starting at 0.8 and increasing to 0.99 after 50 epochs, such that with progressing training the updates are influenced by a larger number of samples than at the beginning.

**Figure 7:**
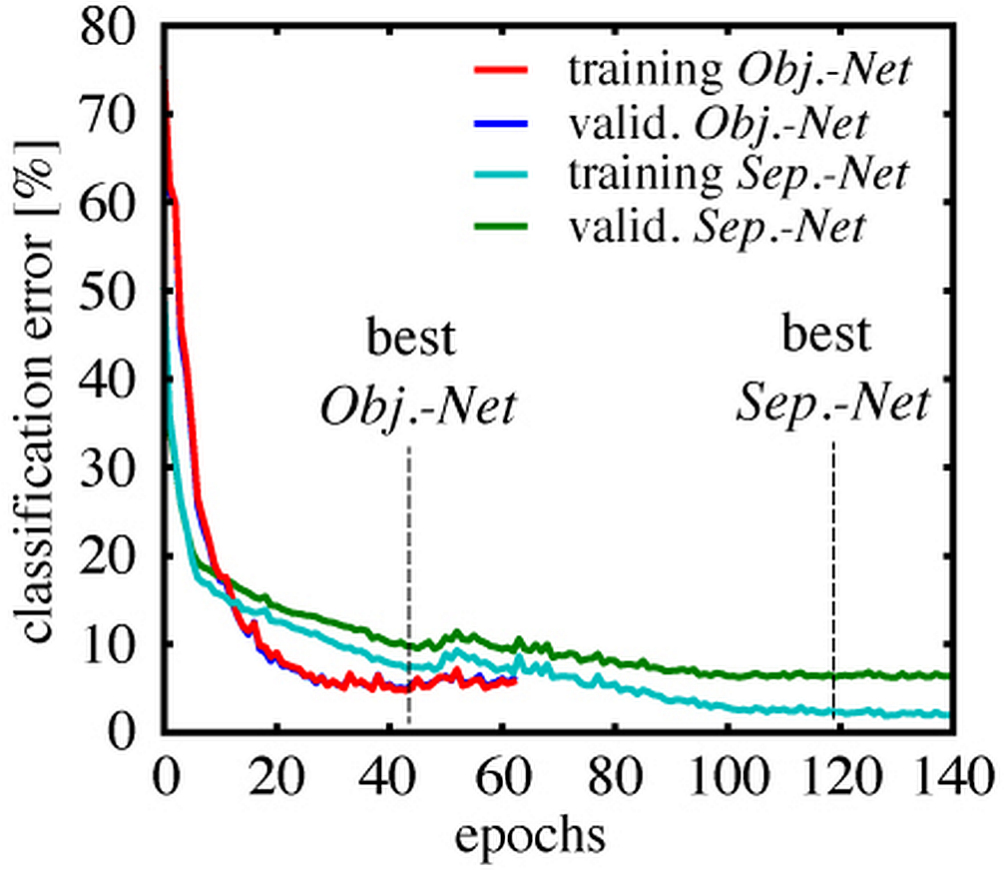
Training and validation error rates during CNN training. Classification error over epochs on a fixed subset of the training data (training error), and on a separate validation set (valid. error), which was not used during training, but used to evaluate the stopping criterion. The *Object-Net* reaches 4.92% validation error after 43 epochs, the *Separator-Net* reaches 6.24% validation error after 119 epochs.

Figure 7 shows the classification error rate as a function of the training duration in epochs. Each class was represented by 5,000 samples in the validation set for the *Object-Net*, and 10,000 for the *Separator-Net*, respectively. The training error was estimated on a fixed subset of the training data (20,000 samples), to get an intuition when overfitting starts. The *Object-Net* achieved the best performance after 43 epochs, with a minimum training error of 4.75% and a minimum validation error of 4.92%. Training of the *Separator-Net* continued until the lowest training error of 2.31% and validation error of 6.24% was reached after 119 epochs. The trained networks were evaluated on a representative test set of 20,000 samples, which shares the same class distribution, but does not overlap with training and validation set. On this test set, the best *Object-Net* achieved an error rate of 4.71%, whereas the *Separator-Net* achieved 5.58%. The CNN models were implemented in Pylearn2 (Goodfellow et al., 2013), a machine learning library built on top of Theano (Bergstra et al., 2010; Bastien et al., 2012).

## Results

### Colon gland segmentation

The grid search resulted in *α* = 10, *β* = 0.95 and *λ* = 0.1 as parameters optimizing the TV segmentation based on the pixel-level Dice coefficient (Eq. (13)). The confidence threshold for foreground and background was determined empirically (in steps of 0.15, starting from 0.5 until 0.95) and fixed to *τ* = 0.65, since for this value there was no influence on the Dice score of the training images.

**Table 1:** Segmentation performance metrics for the *Warwick-QU* dataset used in the *GlaS@MICCAI2015* challenge.

Dataset	PRC	REC	F1	Dice_obj_	HD_obj_
*without* separator refinement					
Training	**0.97**(0.09)	0.67(0.21)	0.78(0.17)	0.81(0.16)	116.89(115.18)
Test A	**0.83**(0.22)	0.60(0.24)	0.67(0.20)	0.70(0.15)	137.44(78.53)
Test B	**0.72**(0.32)	0.55(0.31)	0.57(0.27)	0.62(0.20)	216.40(123.40)
*with* separator refinement					
Training	0.91(0.15)	**0.85**(0.14)	**0.87**(0.12)	**0.88**(0.09)	**61.36**(61.36)
Test A	0.67(0.24)	**0.77**(0.22)	**0.68**(0.20)	**0.75**(0.13)	**103.49**(72.38)
Test B	0.57(0.30)	**0.73**(0.29)	**0.61**(0.27)	**0.65**(0.21)	**187.76**(119.50)

**Notes.**

Metrics are reported as mean and standard deviation (SD), best results are printed in bold. Performance on the training set is reported on all 85 training images. Test set A consists of 60 images, test set B of 20 images. Except for values of the Hausdorff distance (HD_obj_), higher values are superior. Please note that values for F1-score in this table are not directly computed from reported precision (PRC) and recall (REC), but are given as mean (SD) over the individual images in the datasets.

In Table 1, we report mean and standard deviations (SD) of the performance metrics for detection (precision, recall, F1-score), segmentation (object-level Dice), and shape (Hausdorff distance) on the training set, as well as test set A and B provided for the *GlaS@MICCAI2015* challenge. After hole filling and removing blobs with an area less than 500 pixels, all remaining blobs were labeled with unique identifiers before computing the performance measures. The average total runtime for segmenting and classifying a 577 ×  522 image is five minutes using an NVidia GeForce Titan Black 6GB GPU.

#### Influence of the *Separator-Net*

**Figure 8:**
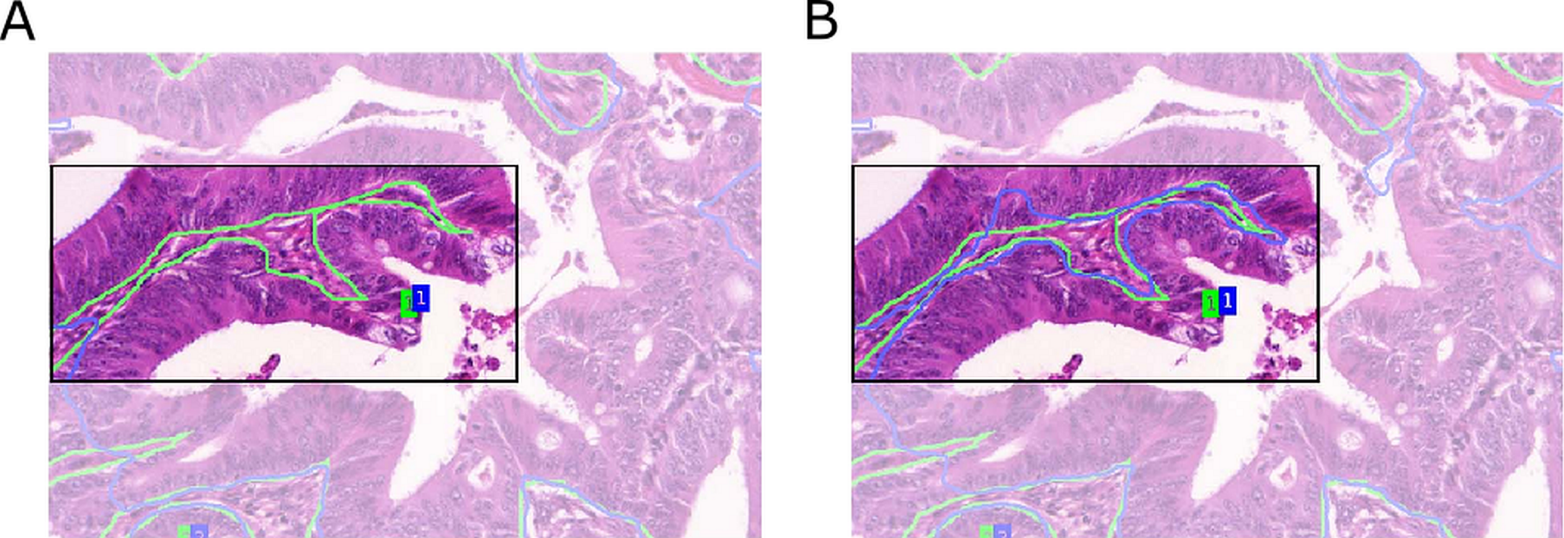
Qualitative segmentation results for a malignant tissue, with and without the use of the *Separator-Net* prediction refinement. Ground truth is outlined in green, the segmentation result in blue, and the region of interest is highlighted. (A) Without prediction refinement, the TV segmentation cannot properly adapt to the irregular glandular shape. The segmentation result including the separator refinement shown in (B) results in a better Hausdorff distance measure.

Compared to predictions arising from the *Object-Net* alone, the segmentation performance improved with separator refinement. This procedure further decreases the Hausdorff measure, since the TV segmentation can better utilize predictions in narrow regions between borders formed by epithelial nuclei. Figure 8 shows a qualitative comparison of segmentation results with and without the *Separator-Net* refinements.

**Figure 9:**
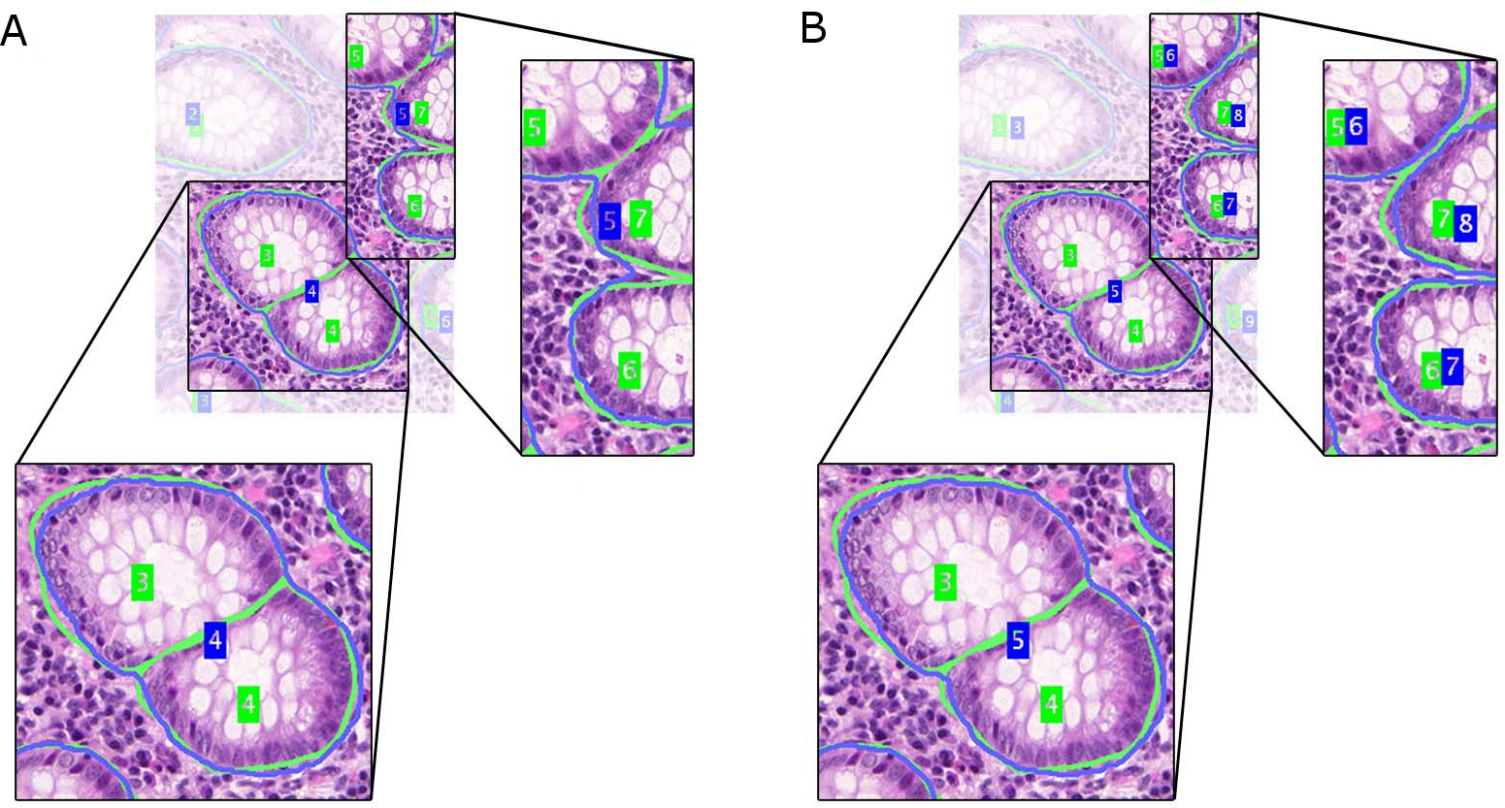
Influence of separator refinement onto splitting adjacent glandular objects. Ground truth is outlined in green, the segmentation result in blue. (A) segmentation without, and (B) segmentation with separator refinement. The separators emphasize the borders of the gland and provide a better input to the TV algorithm with more pronounced predictions, such that adjacent objects can successfully be split. If glands are very close and no significant visual cue for a border can be found, the separator refinement does not have any negative influence on the segmentation.

Malignant cases are harder to segment due to their irregular shape and pathological variations in the tissue. In general, the separator refinement works as expected and allows a better separation of adjacent glands than with *Object-Net* predictions alone. Border regions are more pronounced after the refinement and allow the TV segmentation to better adapt to the true gland borders. However, in cases where two glands are located very closely and there is no significant visual cue for a border or a gland-separating region, the separator does not have any negative influence on the final segmentation result. This is illustrated in Fig. 9.

**Figure 10:**
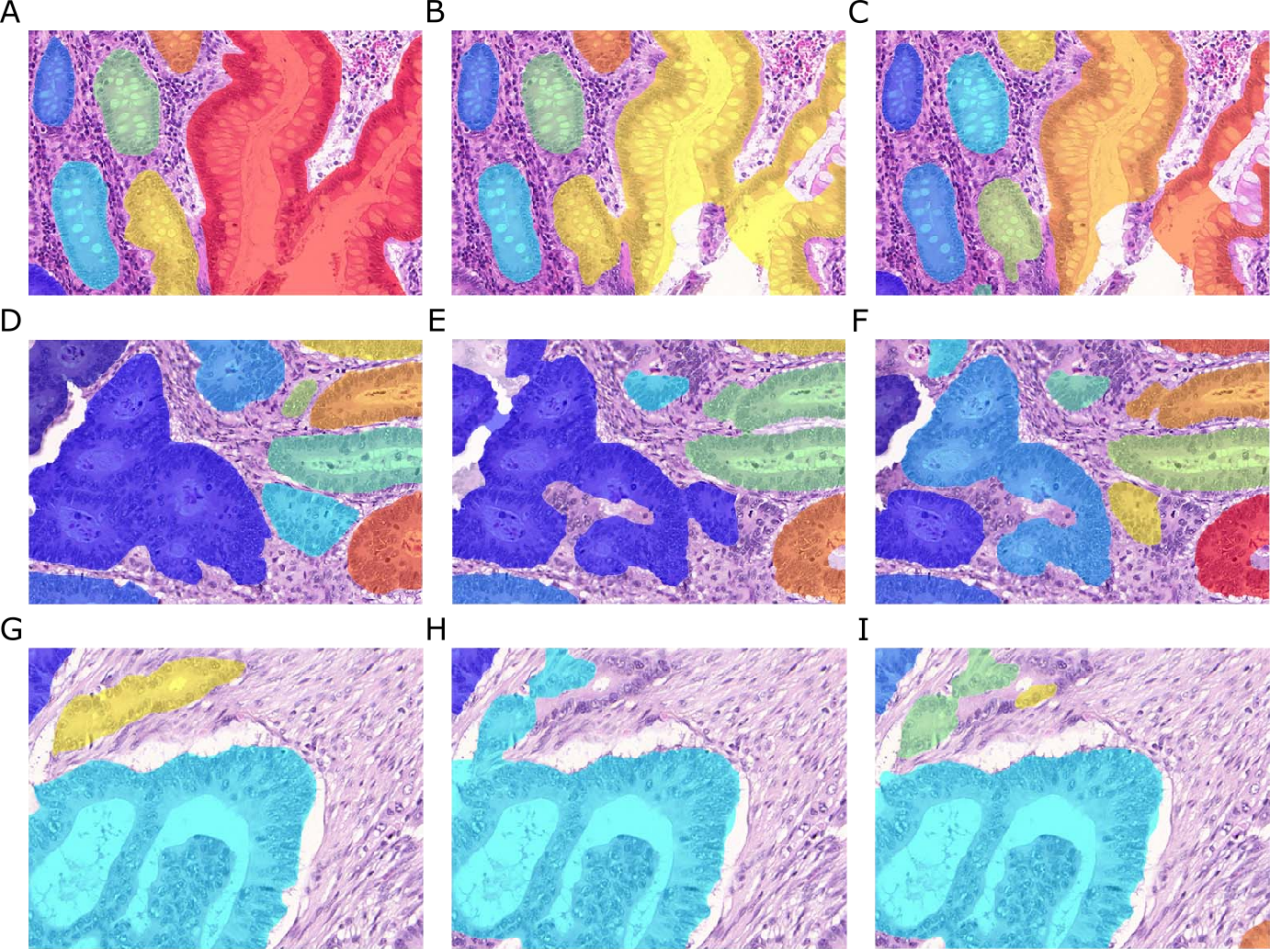
Over-segmentation of the image may be caused by employing separator refinements. Ground truth (A, D, G), segmentation without (B, E, H) and with refinement (C, F, I). Sometimes images get over-segmented when the refinement is applied, causing multiple detections on single gland objects. This may in turn lead to fragmented segmentation of single objects and reduce the true positive rate, as 50% overlap of segmentation and ground truth is required.

As indicated by the precision measures in Table 1, including the separators sometimes leads to an over-segmentation of the image and causes multiple detections on a single object, see Fig. 10. Over-segmentation increases the number of false positives, and at the same time may also decrease the number of true positives, when the overlap between segmentation and ground truth drops below 50%. A possible reason may be that the *Separator-Net* predicts high probabilities for interiors of glands that show highly irregular shape.

Despite having a negative effect on precision, the overall detection performance in terms of F1-score increases when the *Separator-Net* predictions are included. Employing the TV segmentation on the *Object-Net* predictions alone produces fewer, but more extensive segmentation objects. Therefore, the number of false positives is quite low (high precision), while the number of false negatives rises (lower recall). From the object-level perspective, segmentation accuracy and shape similarity measures benefit most from the separator refinements. These findings suggest that—when compared to using only predictions from the *Object-Net*—applying the refinements usually leads to better overall performance.

### Malignancy classification

The confusion matrices shown in Table 2 capture the classification frequency in terms of ground truth (columns) vs. predictions (rows) for benign (*c* = 0) and malignant (*c* = 1) cases. Values for both test sets A and B are given as absolute numbers, the last row contains the total number of samples in the test sets. Table 3 shows performance metrics for both test sets computed from the confusion matrices. The overall classification accuracy (ACC) is in both datasets ≥0.95. Furthermore, it can be observed that our method is very accurate in determining the correct class (F1 >0.88) while keeping the number of *FP* and *FN* samples at bay.

**Table 2:** Confusion matrices for the benignity and malignancy classification in test sets A and B.

		**Ground truth**			
		**Test A**		**Test B**	
	**Class**	benign	malignant	benign	malignant
**Prediction**	benign	32^a^	0^b^	4	1
	malignant	1^c^	27^d^	0	15
		**33**	**27**	**4**	**16**
	**Samples**	**60**		**20**	

**Notes.**

For computation of class-wise tissue classification performance we use the following values:

a True positives (*TP*).

b False positives (*FP*).

c False negatives (*FN*).

d True negatives (*TN*) are not considered in the class-wise metrics, as we report precision and recall for both classes separately in Table 3.

We observe an average conditional probability for each case (Eqs. (7) and (8)) that is classified as either benign or malignant, and this probability can also be interpreted as decision confidence over the entire image. The mean (SD) decision confidence over all cases in test set A was 0.84(0.13) for benign and 0.81(0.11) for malignant, and in test set B 0.74(0.11) and 0.86(0.15), respectively.

**Table 3:** Benignity and malignancy classification performance metrics.

Dataset	Class	ACC	PRC	REC	F1
** Test A**	benign	0.983	1.000	0.970	0.976
	malignant	–	0.964	1.000	0.982
**Test B**	benign	0.950	0.800	1.000	0.889
	malignant	–	1.000	0.938	0.968

**Notes.**

Classification performance is very high for both benign and malignant in both datasets. The relatively low precision (PRC) of 0.800 for benign cases in test set B may be attributed to one *FP* detection with respect to the low total number of four benign samples. Classification accuracy (ACC) is evaluated jointly for benign and malignant cases.

**Figure 11:**
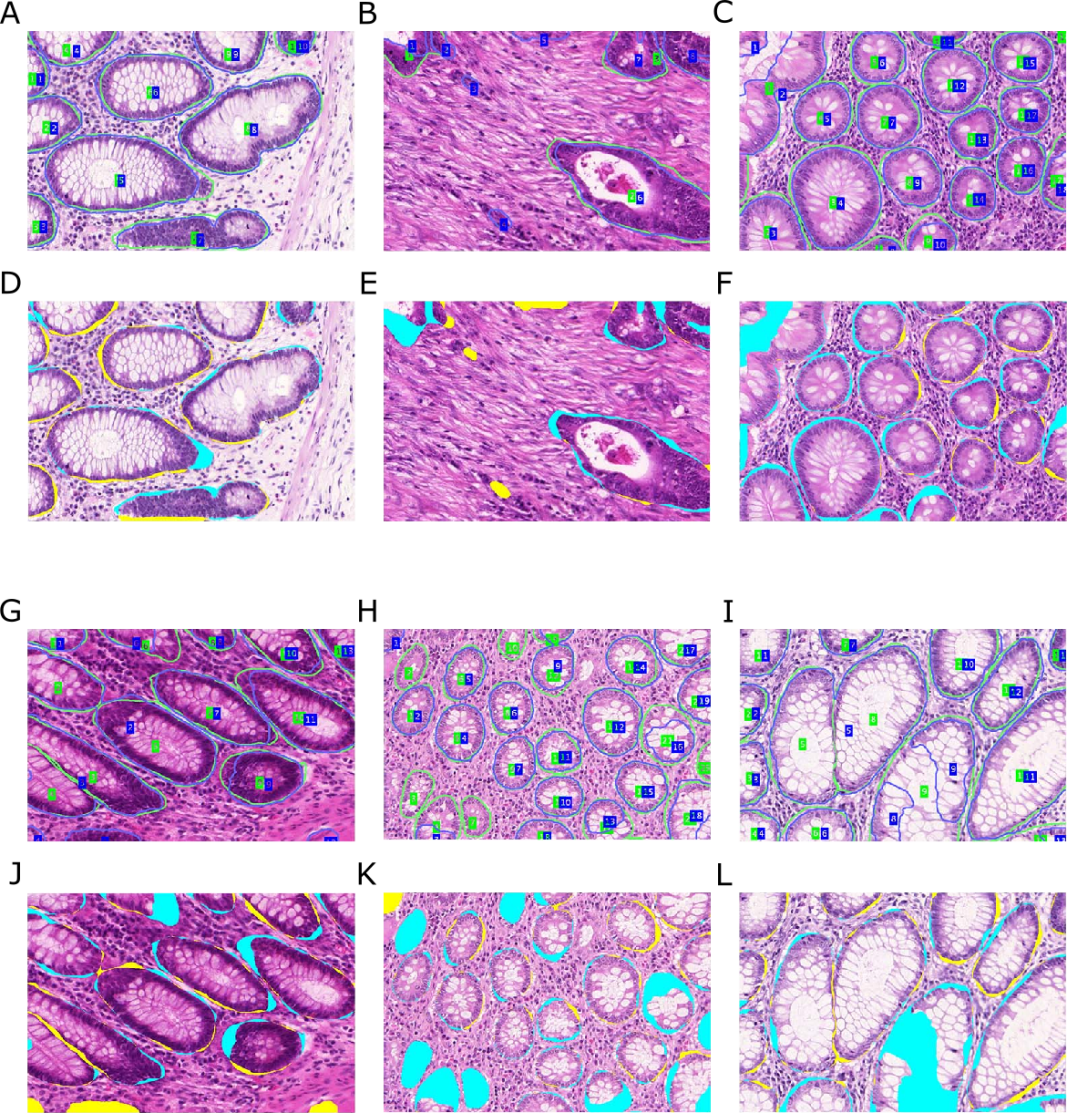
Qualitative segmentation results on images of test dataset A. Segmentation results are illustrated as blue outlines, ground truth in green (A–C, G–I). Differences to the ground truth are shown in (D–F, J–L), where false negative pixels are cyan, and false positive pixels are yellow. (A–F) depict good segmentation results, while (G–L) show different segmentation errors.

### Interpretation

**Figure 12:**
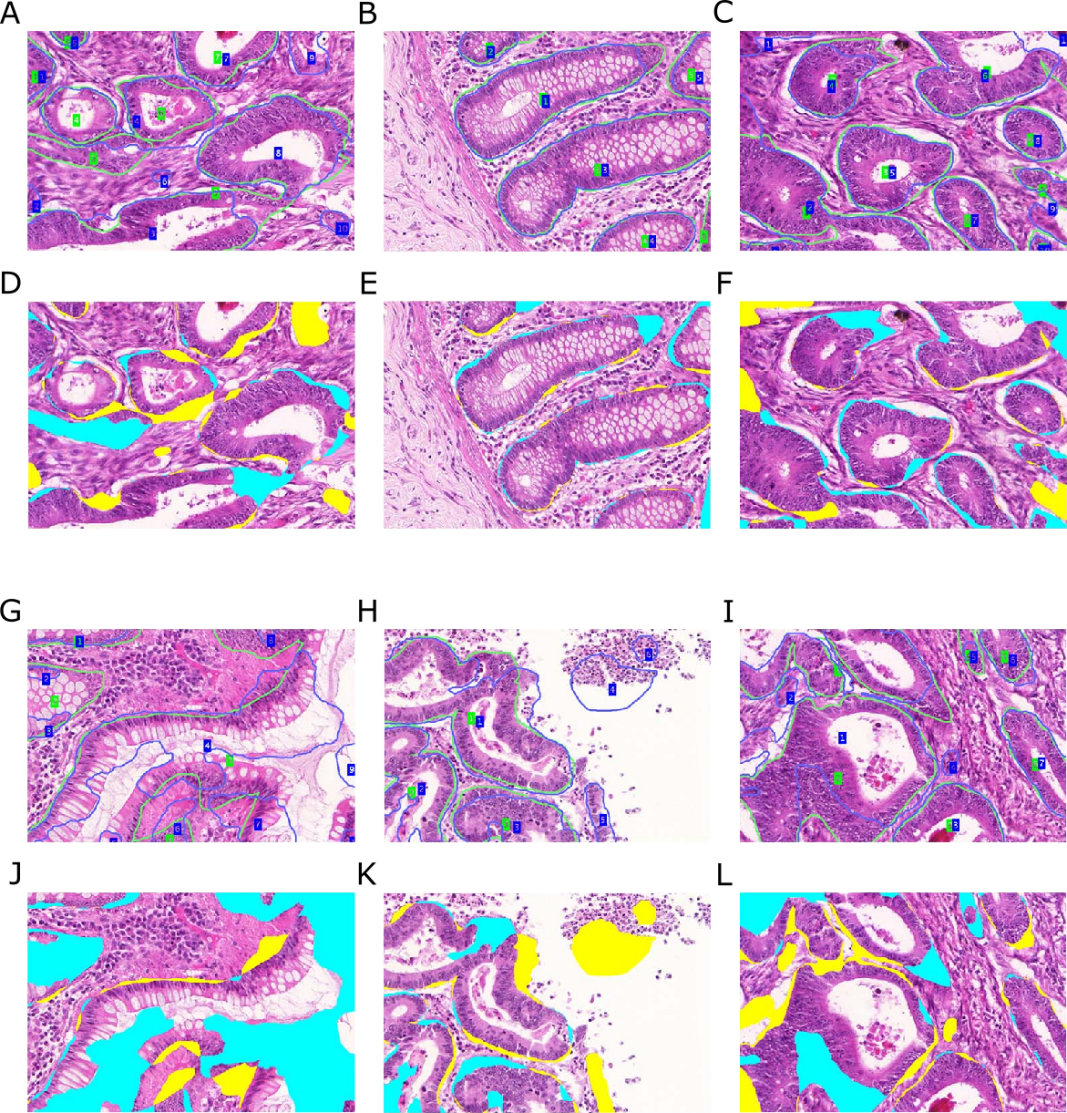
Qualitative segmentation results on images of test dataset B. Segmentation results are illustrated as blue outlines, ground truth in green (A–C, G–I). Differences to the ground truth are shown in (D–F, J–L), where false negative pixels are cyan, and false positive pixels are yellow. (A–F) depict good segmentation results, while (G–L) show different segmentation errors.

Figures 11 and 12 show qualitative results of our approach on test sets A and B, respectively. The best segmentation performance can be achieved on images, where all tissue components can clearly be observed, see e.g., Fig. 12B. Compared to the glandular structure and size, stroma is the most homogeneous tissue region to be found in both benign and malignant cases and seems to contribute significant cues to good segmentation results. We can observe better performance for images, where stroma covers the non-gland image locations (Figs. 11A–11C) than for images, where large lumina are present (Fig. 12G). Furthermore, the biggest advantage of including predictions of the *Separator-Net* can be seen for benign tissue expressing well-defined gland borders.

Some of the images contain a significant portion of non-tissue regions (“true” background), see e.g., the bright part of the image depicted in (Fig. 12H). Since the *Object-Net* was not explicitly trained on different labels for “true” background and lumen, there may be some confusion to classify large lumina as background, and vice versa. Due to the way the *Object-Net* probability maps in Eqs. (1) and (2) are combined, high likelihoods for background where there actually should be lumen results in more false negative pixels in the segmentation, see e.g., cyan parts of the image in Figs. 12G or 11I. However, these errors may also be attributed to the under-representation of these kind of images in the training set.

Independently from the histologic grade of the tissue, extreme variations in gland size cannot be compensated well by our approach. Very small objects may be missed (Fig. 11H), and huge glands may get over-segmented (Fig. 12G). CLAHE preprocessing enhanced detecting objects in images with low staining contrast. However, if the glands are rather small, no clear lumen is visible, and the appearance of the epithelial nuclei in the gland borders is too similar to the adjacent stroma region, the probabilities for foreground are too low to exceed the required confidence threshold (*τ* = 0.65) to be considered as a cue for the TV segmentation. Over-segmentation, on the other hand, may be caused by high probabilities for gland-separating structures within a highly irregularly shaped gland.

## Discussion and Conclusions

This article has introduced an approach for gland segmentation in H&E stained histopathological images of colorectal cancer based on deep convolutional neural networks and total variation segmentation. A contribution of our approach is the use of a second *Separator-Net*, which is trained to resolve particularly difficult cases where different glands are in close contact. We have shown that this refinement improves the segmentation results greatly when compared to pixel-wise classification of gland vs. non-gland alone. This advantage is apparent not only in traditional precision and recall metrics, but also boosts performance scores for detection (F1-score), segmentation (Dice) and shape (Hausdorff). This approach is generally applicable to any biomedical segmentation problem.

Another contribution of our approach is to split the gland vs. non-gland classification problem of the *Object-Net* into a four-class problem, where an additional discrimination between benign and malignant cases is learned. This also allows a categorization of the whole image, depending on whether the majority of tissue is classified as benign or malignant, and in addition provides a confidence value for this decision, which is of promising accuracy above 95% on our test cases. Potentially, this approach also allows to distinguish between even more states of the tissue, e.g., more detailed histologic grades.

In comparison to previous work on gland segmentation (Wu et al., 2005; Naik et al., 2008), our approach does not require prior knowledge on the shape and structure of glands, because this is learned from labeled data. Of course such data, and in particular reliable ground truth information is scarce, and datasets such as the one published for the *GlaS@MICCAI2015* challenge can only provide a starting point. To enable an algorithm that can be used in a general medical diagnostic setting, it would be necessary to have significantly larger datasets from which different subtypes of benign and malignant gland types could be learned. However, model-based approaches might experience similar problems, since prior knowledge on the appearance of glands might not accurately describe the many potential forms of malignant tissues that could be observed. A potential solution might be the use of hybrid models, which combine learned models with expert knowledge for specific applications.

Finding intact gland borders is a key requirement of the approach presented by Fu et al. (2014). They showed the applicability of their method to pre-invasive and well or moderately differentiated cancerous tissue, where the gland borders can still be identified. Since this prerequisite cannot be expected for poorly differentiated (high-grade) tumorous tissue, their method would likely work on only a subset of images in the *Warwick-QU* dataset. Naturally, any segmentation method will benefit from finding intact gland borders in the images, however, since our approach does not rely on explicitly finding gland border cell nuclei, this issue may be neglected. Furthermore, since we are using a separate prediction for regions close to gland borders in the case of segmenting very close objects, our proposed method remains more flexible for multiple histologic tumor grades.

Localization of gland lumen as seed regions is often considered as a first step in most of the related work. The underlying assumption is that lumen regions are rather homogeneous in their texture and intensities, and are spatially constrained by the epithelial nuclei in the gland borders. The MSER detector (Matas et al., 2002) could be employed to automatically create candidate lumen regions, but such an approach has not yet been reported in the literature. However, as previously concluded (Nguyen, Sarkar & Jain, 2012), this does not work for occluded lumina, since candidates are created based on an intensity homogeneity assumption. It would be interesting to see whether this problem could be avoided by learning the appearance of lumina from local texture, where the problem of occluded lumina can also be addressed. Nevertheless, our approach does not require such an initial lumen detection step, but learns lumen regions as part of an entire gland.

Moreover, the localization of epithelial nuclei plays an important role in delineating gland borders—in particular when lumina are used as seed regions (Naik et al., 2008; Nguyen, Sarkar & Jain, 2012; Rashid et al., 2013; Sirinukunwattana, Snead & Rajpoot, 2015). Most of the existing approaches treat nuclei detection as an unsupervised classification problem using *k*-means on the input color space. However, this depends strongly on the staining of the section, which may vary greatly, even when it is processed in the same histology lab. A very recent work (Kainz et al., 2015) presented an alternative learning-based method to detect cell nuclei in histopathology images using a regressor that learns to predict, for each image location, the distance to the closest cell from image features. Supervised learning could be employed to more robustly locate gland border nuclei and overcome existing problems in clustering-based strategies, where a subjectively defined amount of additional prior knowledge is required to achieve proper results.

Despite the fact that previous work resulted in a large variety of methods on gland segmentation, a vast majority of contestants at the *GlaS@MICCAI2015* challenge employed deep learning methods. A major reason of this popularity is certainly the recent groundbreaking success of deep learning methods in computer vision and pattern recognition (Krizhevsky, Sutskever & Hinton, 2012; Cireşan et al., 2013), and the availability of GPU hardware and learning frameworks like Torch (Collobert, Kavukcuoglu & Farabet, 2011), Caffe (Jia et al., 2014) and Theano/Pylearn2 (Bergstra et al., 2010; Bastien et al., 2012; Goodfellow et al., 2013) that reduce the training time for massive neural networks with backpropagation to acceptable time scales. Thirteen teams participated in the on-site contest and were included in the final ranking of the contest (Sirinukunwattana et al., 2017), contributing a total of 19 algorithms. The most successful approaches all used some form of deep learning, whereas the approaches that did not were ranked towards the end. The leading method employed a variant of fully convolutional networks (FCN) (Long, Shelhamer & Darrell, 2015). Our approach showed very promising results on two test problems, and exhibited aspects that were not used by other teams, such as the two-network approach and the distinction between benign and malignant tissue as a tool to improve segmentation performance. Although FCN outperformed our more classical CNN architecture in the *GlaS@MICCAI2015* contest, our approach ranked overall 9th of 19 competing algorithms (some were different variants from the same team), with our results for malignant glands only even ranking 8th out of 19. For the final paper summarizing the challenge (Sirinukunwattana et al., 2017), we were among the six best teams chosen for being included when presenting the overall segmentation results.

Regarding an improvement in performance, it is likely that a combination of our approach with other successful strategies, such as the use of FCN instead of standard CNN, and the use of larger network architectures, would result in an even better gland segmentation performance. This is expected to result in more robust pixel-wise predictions and hence will simplify the problem for the subsequent TV segmentation. It is further interesting that recently, our idea of splitting gland segmentation and edge based segmentation was picked up in the work of Xu et al. (2016), implementing both in a single CNN framework. The final ranking as well as the test set performance results of other algorithms participating in this challenge are available online at the contest website and summarized in Sirinukunwattana et al. (2017).

The very different approaches applied by different groups participating in the contest suggest that a combination of the introduced approaches has the potential to improve the quality of automatic segmentation even further. In particular, we did not experiment with complex morphological operations to enhance the segmentation results, apart from simple hole filling and blob removing. Post-processing could also potentially remove several false positives after TV segmentation, e.g., by extracting different features from the segmented regions and employing a final classifier. Similarly, we think that our approach, in particular the use of *Separator-Net*s, could become a very useful ingredient aiding the performance of other presented solutions. Furthermore, our method is not specific to the colon gland data used in the *GlaS@MICCAI2015* challenge, and can be evaluated for other segmentation tasks given publicly available datasets.

## Additional Information and Declarations

### Data Availability

The following information was supplied regarding data availability: The trained models of the deep convolutional nets are available at: https://github.com/pkainz/glandsegmentation-models. Input data and evaluation scripts are provided by the *GlaS@MICCAI2015* challenge website http://www2.warwick.ac.uk/fac/sci/dcs/research/tia/glascontest.
